# ChatGPT-Enhanced ROC Analysis (CERA): A shiny web tool for finding optimal cutoff points in biomarker analysis

**DOI:** 10.1371/journal.pone.0289141

**Published:** 2024-04-10

**Authors:** Melih Agraz, Christos Mantzoros, George Em Karniadakis

**Affiliations:** 1 Department of Statistics, Giresun University, Giresun, Turkiye; 2 Department of Medicine, Boston VA Healthcare System, and Department of Medicine, Beth Israel Deaconess Medical Center, Harvard Medical School, Boston, MA, United States of America; 3 Division of Applied Mathematics, Brown University, Providence, Rhode Island, United States of America; 4 School of Engineering, Brown University, Providence, Rhode Island, United States of America; International University of Health and Welfare, School of Medicine, JAPAN

## Abstract

Diagnostic tests play a crucial role in establishing the presence of a specific disease in an individual. Receiver Operating Characteristic (ROC) curve analyses are essential tools that provide performance metrics for diagnostic tests. Accurate determination of the cutoff point in ROC curve analyses is the most critical aspect of the process. A variety of methods have been developed to find the optimal cutoffs. Although the R programming language provides a variety of package programs for conducting ROC curve analysis and determining the appropriate cutoffs, it typically needs coding skills and a substantial investment of time. Specifically, the necessity for data preprocessing and analysis can present a significant challenge, especially for individuals without coding experience. We have developed the CERA (ChatGPT-Enhanced ROC Analysis) tool, a user-friendly ROC curve analysis web tool using the shiny interface for faster and more effective analyses to solve this problem. CERA is not only user-friendly, but it also interacts with ChatGPT, which interprets the outputs. This allows for an interpreted report generated by R-Markdown to be presented to the user, enhancing the accessibility and understanding of the analysis results.

## Introduction

A diagnostic test is a medical or statistical procedure used to assess the likelihood or propensity of an individual to develop a specific disease. These tests, particularly those with binary outcomes, are evaluated based on performance measures such as sensitivity and specificity. A key tool in this evaluation is the Receiver Operating Characteristic (ROC) curve, which visually represents the relationship between the true positive rate (TPR or sensitivity) and the false positive rate (FPR, equivalent to 1-specificity). The area under the ROC curve (AUC) is a well-acknowledged measure of a test’s accuracy, offering insightful interpretations of its performance [1].

The concept of the ROC curve, essential for evaluating diagnostic tests and classification models, originated in radar technology during World War II and was later applied in the 1950s for signal detection [2, 3]. Building on this historical foundation of ROC curve analysis in evaluating diagnostic tools, contemporary approaches have further refined how these tools are utilized, particularly in the crucial aspect of establishing cutoff values for diagnostic tests. When it comes to establishing a cutoff value in diagnostic tests, two primary statistical approaches are employed: biomarker-oriented and outcome-oriented [4]. The biomarker-oriented method focuses on the biomarker itself, using statistics like mean, mode, or specific percentiles for determining the cutoff, without considering the response variable. In contrast, the outcome-oriented method examines the relationship between the biomarker and the response variable, often resulting in more precise cutoffs. Due to its higher accuracy, the outcome-oriented approach is widely recommended for determining cutoff values in diagnostic tests [5].

There are several tools available, both commercial and open-source, for calculating ROC curves and determining optimal cutoff points. Some commonly used commercial tools for this purpose include SPSS, MedCal, Stata, and Minitab. Additionally, there are powerful and freely available programming languages like R and Python that can be utilized for these tasks. R, in particular, is well-known for its extensive capabilities in statistical analyses and offers numerous packages specifically designed for calculating ROC curves and determining optimal cutoff points. These include ROCR [6], plotROC [7], pROC [8], OptimalCutpoints [9], precrec [10], and ROCit [11]. In addition, there are web-based tools powered by the shiny framework for ROC curve analysis and cutoff value calculation. For instance, easyROC is a shiny-based web tool for ROC curve analysis [12] that calculates ROC curves, cutoff points, and sample sizes. Another shiny tool, the cutoff finder [13], calculates ROC curves and cutoff values, and additionally enables users to conduct survival analysis.

Processing the data in ROC curve analysis, calculating the statistical results of the biomarker, displaying them graphically, and calculating the cutoff values by performing the ROC curve analysis is always difficult for those who are not familiar with the R programming language, especially for medical doctors. Therefore, in this study, a user interface web tool with three different versions are developed for those who do not know programming languages or are looking for faster solutions in ROC curve analysis. Additionally, in version 1 and 2, a key feature of this tool is its ability to automatically generate interpretations of the outputs, enhanced by ChatGPT technology. We are providing the ChatGPT-Enhanced ROC Analysis (CERA) tool which performs ROC curve analysis and optimal cutoff values with a shiny user-interface web tool. Additionally, in version 3, users have the option to upload their own data or select from example datasets, input a prompt, and observe as the tool not only generates R code but also provides visualizations of this code.

CERA is a tool that establishes an API connection with ChatGPT, enabling the interpretation of results derived from a Shiny application. This allows users without coding knowledge to perform ROC and data science analysis. As ChatGPT itself discloses, it does not guarantee 100% accurate results. This limitation is acknowledged in ChatGPT’s interface statement: ‘ChatGPT may produce inaccurate information about people, places, or facts.’ After analyzing data and calculating optimal cutoff points, CERA prepares a report in version 1 and 2. Acknowledging the potential for inaccuracies from ChatGPT, we incorporate a disclaimer about possible misinterpretations at the beginning of each report generated by the CERA tool. We also cannot guarantee the absolute accuracy of reports prepared using CERA. However, in comparisons of reports generated from the same dataset, with identical biomarkers, outputs, and cutoff value methods, the results were largely accurate. As is widely known, ChatGPT, released in November 2022, is a novel LLM tool. We anticipate that as ChatGPT continues to develop, it will yield increasingly accurate results. Consequently, the accuracy of results provided by the CERA tool is also expected to improve in tandem with ChatGPT’s evolution. In this paper, we explore ROC curve analysis in the Materials and Methods section, emphasizing its workflow within the CERA tool. In the Results section, we delve into the performance and interpretation of the CERA tool’s outputs, including the ROC plot, biomarker statistics, and the generated report enhanced by ChatGPT. The Conclusion section briefly synthesizes the insights gained from the tool, highlighting its novelty and potential for future enhancements in data analysis and interpretation.

## Materials and methods

**Fig 1:**
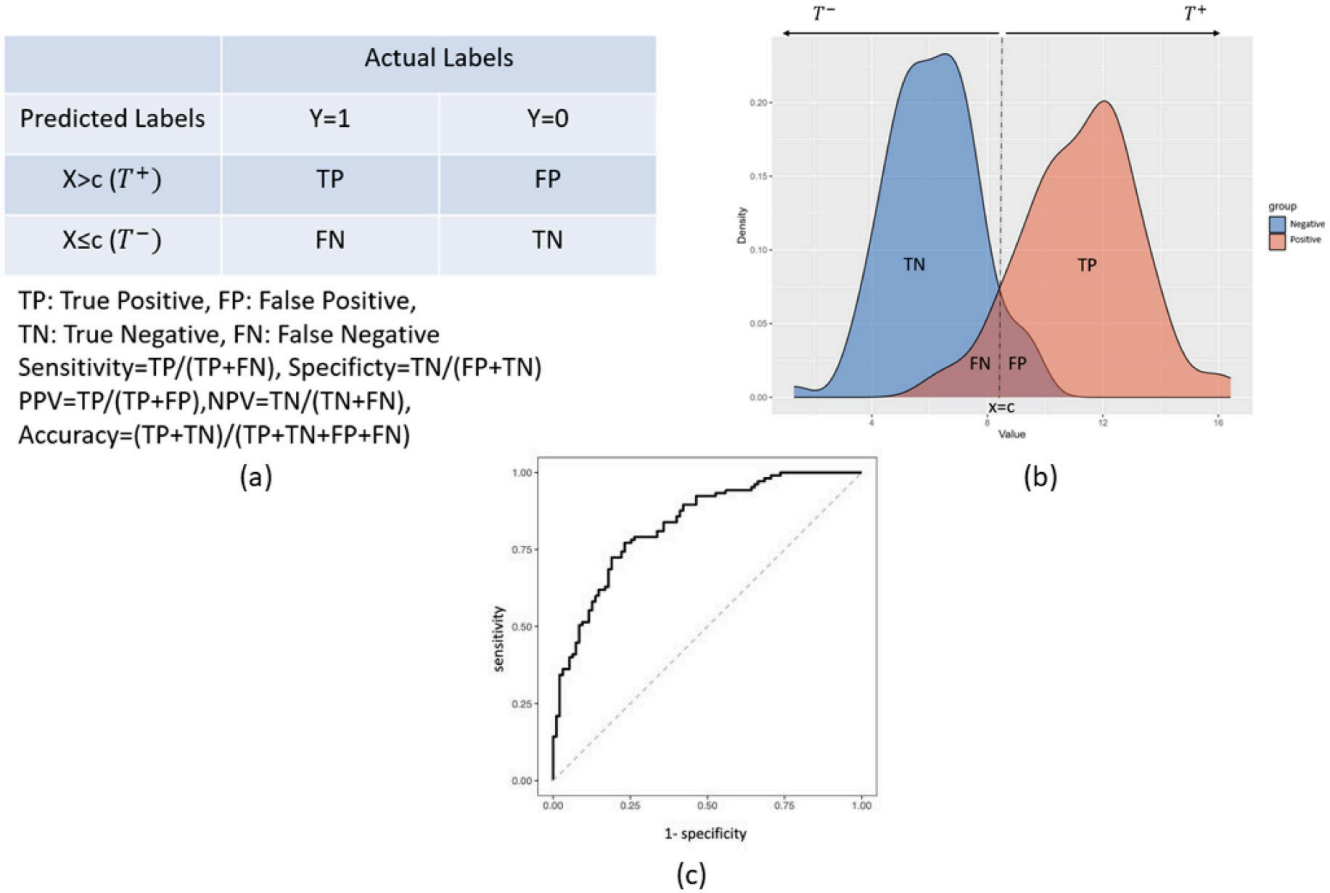
Overview of the ROC analysis process and performance metrics. (a) 2x2 classification table illustrating the performance of the classifier. The table demonstrates the different types of predictions made by the classifier based on the chosen cutoff value, including true positives (TP), false positives (FP), true negatives (TN), and false negatives (FN). (b) Hypothetical distributions of decision-making, demonstrating overlap between the positive and negative labels. The overlapping regions represent the potential for misclassification by the classifier. (c) ROC curve created from two overlapping distributions as in Fig (b), highlighting the performance of the classifier at various cutoff points. The ROC curve gives a visual depiction of the classifier’s discriminating power by demonstrating the trade-off between sensitivity and specificity for various threshold values.

ROC curve is a graphical representation extensively utilized to illustrate the discriminatory accuracy of a diagnostic test or marker between two distinct groups. This technique has garnered significant attention owing to its effectiveness in showcasing the marker’s discriminatory accuracy. Essentially, the ROC curve offers a visual framework for comprehending the proficiency of a specific marker in distinguishing between two disparate populations. Diagnostic tests categorize individuals into two groups, such as positive/negative (Fig 1a), and there is always a potential for error, given that these two groups may overlap. Let us assume an example that two groups, positive and negative, overlap as depicted in Fig 1b, and these groups are distinguished by the threshold *c* as shown in Fig 1a; we need to elucidate the types of errors that might occur. As can be observed in Fig 1b, values greater than the *c* cutoff are predicted to be positive (*T*+), while smaller values are predicted to be negative (*T*−). However, the region we denote as FP (Type I error) is actually negative, but it is predicted as positive. The remaining predictions, which are correctly identified as positive, are denoted as TP. Similarly, values smaller than *c* are correctly predicted as negative (TN), while incorrect predictions are labeled as FN (Type II error). If we can generate tables (as shown in Fig 1a) for each candidate threshold, and calculate the sensitivity versus (1-specificity) values, we can create the figure shown in Fig 1c. This figure displays the ROC curve for two overlapping groups. As indicated in Fig 1c, the diagnostic test appears to be a more effective discriminator than a random guess; it is certain that it is higher than 50%. Sensitivity and specificity levels higher than 90% and ideally higher than 95% are considered clinically appropriate and useful.

### Datasets

In this study, we showcase the effectiveness of our tool using both public and simulated datasets. As the dataset consists of publicly available and simulated data, ethics committee approval is not required.

#### Aneurysmal subarachnoid hemorrhage (aSAH) dataset

Aneurysmal subarachnoid hemorrhage (aSAH) is a form of hemorrhagic stroke that comes with considerable health complications and has a death rate close to 50% [14–16]. This aSAH data [17], utilized as an example dataset in the CERA tool and available in the pROC package [8], comprises 113 individuals with aneurysmal subarachnoid hemorrhage. It includes 6 explanatory variables and 1 binary outcome variable, “aneurysmal_subarachnoid_hemorrhage” (Good/Bad). The explanatory variables are Glasgow outcome score at 6 months (GOS6), gender, age, world federation of neurological surgeons (WFNS), S100*β*, and NDKA.

#### PIMA Indian dataset

Type 2 diabetes mellitus (T2DM), a prevalent chronic metabolic disorder, results from a complex interplay of genetic, epigenetic, and environmental risk factors [18]. Another example dataset available in the CERA tool is the PIMA Indian dataset [19], sourced from Kaggle [20], collected by National Institute of Diabetes and Digestive and Kidney Diseases (NIDDK). This is followed study from 1982 through 2007 and the dataset is particularly relevant as it shows the high prevalence of type 2 diabetes among women of PIMA Indian heritage who are at least 21 years old. It consists of eight variables and a target variable, with a total of 768 observations. The features included in the dataset are as follows: pregnancies (number of times pregnant), glucose (plasma glucose concentration), blood pressure (diastolic blood pressure, mm Hg), sin thickness (triceps skin fold thickness in mm), insulin (mu U/ml), BMI, diabetes pedigree function, and Age (years). The target variable, namely “Diabetes”, for binary classification takes the values of 0 or 1, where 0 indicates a negative test result for diabetes, and 1 indicates a positive test result.

#### Wisconsin breast cancer dataset

Breast cancer is the most common type of cancer affecting women in terms of incidence [21]. In this tool, we use the Wisconsin Breast Cancer Dataset as an example dataset [22], available at UCI Machine Learning repository [23], which comprises 9 features, Clump Thickness, Uniformity of Cell Size, Uniformity of Cell Shape, Marginal Adhesion, Single Epithelial Cell Size, Bare Nuclei, Bland Chromatin, Normal Nucleoli, Mitoses, along with the target variable “Breast_Cancer”. This dataset contains a total of 699 observations. The dataset provides valuable information for conducting analysis and exploring the relationship between the features and the presence of breast cancer output with 2 for benign, 4 for malignant.

#### Non-alcoholic fatty liver disease (NAFLD) simulated dataset

Nonalcoholic fatty liver disease (NAFLD), recognized as the most common cause of liver disease [24], is frequently assessed using the hepatic steatosis index (HSI) as a screening tool [25]. Consequently, we simulate a synthetic dataset for NAFLD, consisting of 300 patients, with a binary response variable (NAFLD/non-NAFLD, labeled as 1/0). In this simulated dataset, data labeled ‘1’ (indicating NAFLD) has a mean HSI of 55 with a standard deviation of 10. Conversely, data labeled ‘0’ (indicating non-NAFLD) has a mean HSI of 40, also with a standard deviation of 10.

### Main elements of the tool

**Fig 2:**
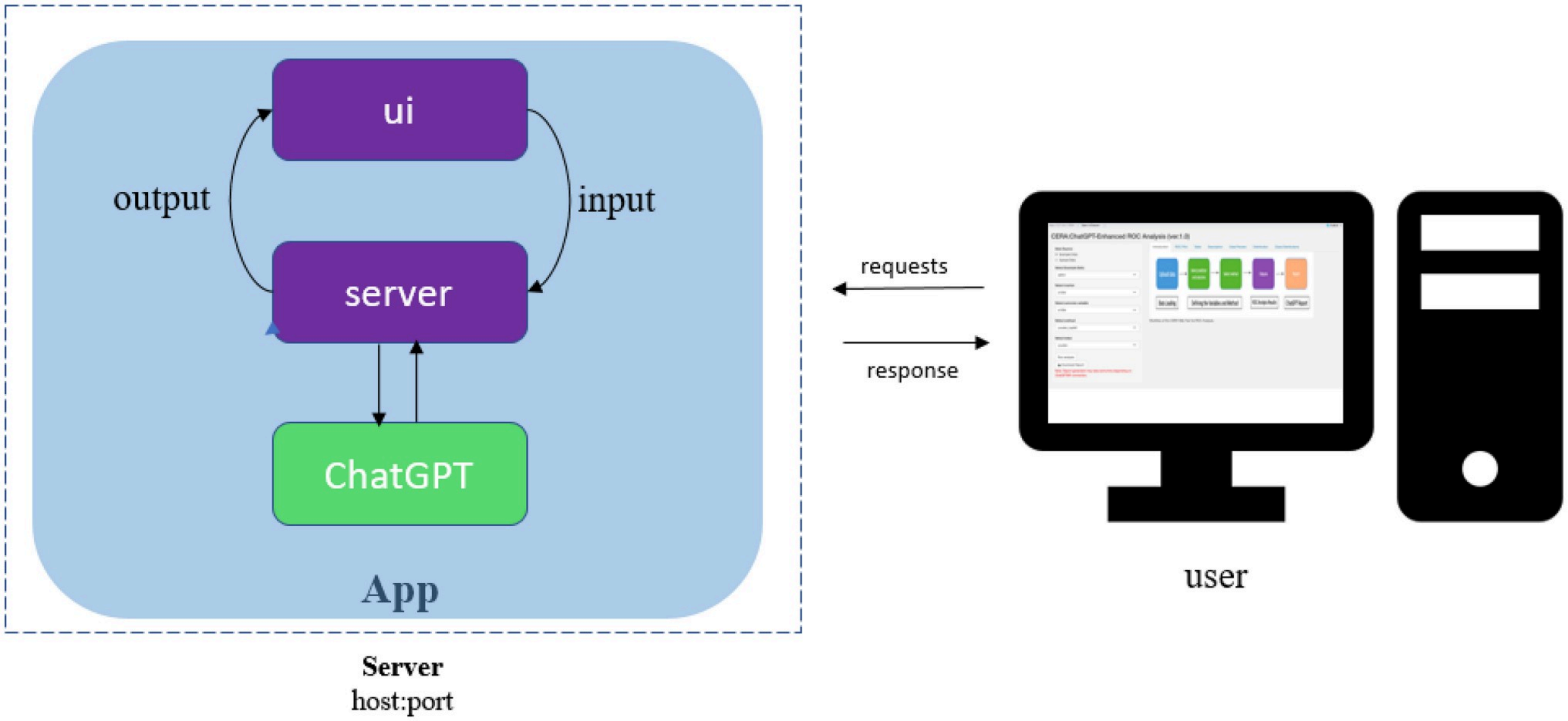
A shiny workflow diagram illustrating the UI, server, and ChatGPT API connection. The diagram visually represents a dynamic workflow demonstrating the interactions between the user interface (UI), the server, and the ChatGPT API within the context of data analysis and reporting. The CERA tool enables users to upload their data, select markers, and choose appropriate methods for analysis. This step signifies the user’s input and customization of the analysis process. Once the necessary inputs are provided, the user can proceed by clicking on the “Run analysis” button. This action triggers the tool to perform the ROC analysis. Upon completion, the user can proceed by clicking on “Download Report” to establish a connection with the ChatGPT API in version 1 and 2. This step allows the user to receive a report generated by ChatGPT, which includes interpretations of plots and tables. CERA version 3 has a similar workflow diagram, but it uses ChatGPT solely for answering user questions and does not generate reports interpreted by ChatGPT.

We are using the shiny tool to convert the R-generated ROC curve analysis to a user interface web tool. A typical shiny application is composed of two main elements represented in Fig 2: (i) **user interface (UI)** that constructs the visual appearance, and (ii) **server** that contains instructions for executing and updating the objects presented in the UI. In addition to that, the proposed CERA tool has a **ChatGPT API connection** as represented in Fig 2. In the shinny app, the UI determines the visual appearance and layout of the web application. The UI code defines the organization of the application, including the various input elements (e.g., sliders, text boxes) and output components (e.g., charts, tables) that users interact with. On the other hand, the server is responsible for generating dynamic outputs based on user inputs and updating them in real-time. It handles the computational and data processing tasks of the application. When the user interacts with the UI, the server processes the input data, performs the necessary calculations or manipulations, and sends the computed results back to the UI for display. In this study, we have incorporated the ChatGPT API connection using the httr [26] library to provide interpretations for the ROC curve analysis results. The gpt-3.5-turbo version is utilized. The connection to the API is established when the user clicks on the “Download Report” button in version 1 and 2, and the report is generated based on the results of the ROC curve analysis. In version 3, the integration with ChatGPT is activated when users click the ‘Run’ button after entering their prompt into the textbox. The report generated in version 1 and 2 consists of three sections: Introduction, Results (with subsections for data quality checking, ROC plot, and performance measures), and Conclusion. The API connection prompts are fixed and not user-modifiable in version 1, but in version 2, user can write their own prompts to generate report. In both version 1 and 2 as described in the following parts, the CERA tool establishes an API connection for each section and subsection of the report, resulting in a total of five API connections for each report preparation. Furthermore, to make the tool more flexible, we developed the version 3. In this version, user inputs are converged to code/text and it is converted to plot if needed.

In version 2 of the CERA tool, users can create custom prompts. Additionally, version 1 provides the capability for the automatic generation of a ChatGPT report. In version 2, we have included example prompts as represented under the Workflow steps for version 2 subsection, allowing users to view examples similar to those used in version 1. Although we can showcase a few examples here, the complete code is accessible on our GitHub page. For instance, when interpreting performance measures displayed under the ‘Table TabPanel’, we suggest this default prompt:

INPUT PROMPT: “Interpret the results of the ROC biomarker analysis based on the following data: Threshold: 45.326, Sensitivity: 0.733, Specificity: 0.893, and Accuracy: 0.813. Start your answer with the following: ‘Table 1 shows the performance…’”
CHATGPT OUTPUT: “Table 1 shows the performance measures of the ROC analysis. The ROC biomarker analysis at a threshold of 45.326 yielded promising results. The sensitivity of 0.733 indicates that the biomarker correctly identified 73.33% of true positive cases, suggesting its effectiveness in detecting the condition of interest. Additionally, the specificity of 0.893 signifies that the biomarker exhibited a high ability to correctly classify true negative cases, minimizing the chances of false positives. This indicates its precision in ruling out individuals who do not have the condition. The overall accuracy of 0.813 implies that the biomarker performed well in discriminating between positive and negative cases, with an 81.33% correctness rate. These results suggest that the biomarker shows potential as a valuable diagnostic tool, particularly when balanced sensitivity and specificity are crucial for the clinical or research context in which it is being applied.”

**Table 1:** Performance measures of ROC curve analysis generated by CERA tool. The table represents the performance measures of ROC curve analysis for the pexample prompt, as shown in the Table Tab panel of the CERA tool. The results indicate the following metrics: threshold: 45.33, Sensitivity: 0.73, Specificity: 0.89, Positive Predictive Value (PPV): 0.87, Negative Predictive Value (NPV): 0.77, Accuracy: 0.81, Area Under the Curve (AUC): 0.87, Confidence Interval (CI) of Lower Boundary (LB)-Upper Boundary (UB): 0.83-0.91.

Threshold	Sensitivity	Specificity	PPV	NPV	Accuracy	AUC	CI: LB-UB	
45.33	0.73	0.89	0.87	0.77	0.81	0.87	0.83	0.91

For another example, here’s a sample Conclusion section prompt:

INPUT PROMPT: “Write a Conclusion section. In this research, we conducted a Receiver Operating Characteristic (ROC) curve analysis to determine the optimal cut-off points for the “HSI” biomarker in relation to “NAFLD”. The ROC curve graphically represents the trade-off between sensitivity and specificity for a classifier at different thresholds. The area under the ROC curve (AUC) allows us to assess the diagnostic accuracy of the “HSI” biomarker in predicting “NAFLD”. Our primary goal is to identify the most appropriate cut-off values for the “HSI” biomarker, optimizing early detection and diagnosis of “NAFLD”. For your conclusion, consider integrating the following key findings: the AUC is 0.8703, and the recommended threshold for the “HSI” biomarker is 45.326.”
CHATGPT OUTPUT: “In conclusion, this research employed Receiver Operating Characteristic (ROC) curve analysis to ascertain the ideal cut-off point for the “HSI” biomarker concerning “NAFLD.” The ROC curve provided a visual representation of the sensitivity-specificity trade-off across various thresholds, with the area under the ROC curve (AUC) serving as a pivotal metric for evaluating the “HSI” biomarker’s diagnostic accuracy in predicting “NAFLD.” Our findings revealed a commendable AUC of 0.8703, signifying the robust predictive capacity of the “HSI” biomarker. Importantly, the recommended threshold for the “HSI” biomarker, determined to be 45.326, holds significance for optimizing the early detection and diagnosis of “NAFLD.” These results underscore the potential clinical utility of the “HSI” biomarker as a valuable tool in the timely identification of individuals at risk for “NAFLD,” ultimately contributing to more effective healthcare interventions and improved patient outcomes”.

**Fig 3:**
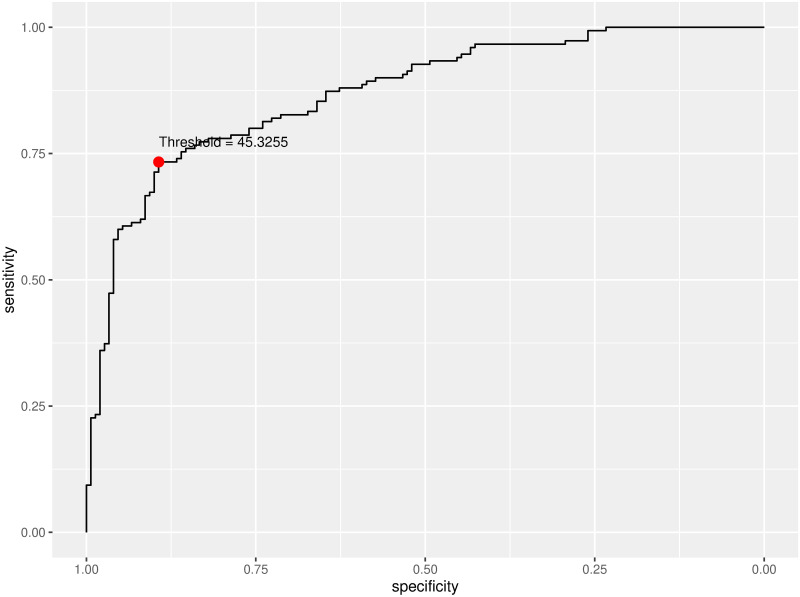
ROC curve generated by CERA tool. The curve plots sensitivity against specificity for different thresholds. The red dot represents the optimal threshold at a value of approximately 45.326, chosen to balance true positive and true negative rates effectively.

In addition to the output generated by ChatGPT, the CERA tool provides Fig 3 and Table 1, which ChatGPT subsequently utilizes as input. Fig 3 depicts a ROC curve, employed to evaluate the performance of a diagnostic test for NAFLD. The curve illustrates sensitivity on the y-axis against specificity on the x-axis across various threshold levels. A point marked in red designates the optimal Youden’s threshold value, selected to enhance the test’s capability to accurately identify both true positives and true negatives. In this instance, the threshold HSI is approximately 45.326.

Table 1 presents the performance measures of the ROC curve analysis with the following metrics: threshold at 45.33, Sensitivity of 0.73, Specificity of 0.89, Positive Predictive Value (PPV) of 0.87, Negative Predictive Value (NPV) of 0.77, Accuracy of 0.81, Area Under the Curve (AUC) of 0.87, and a Confidence Interval (CI) for the Lower Boundary (LB) to Upper Boundary (UB) ranging from 0.83 to 0.91.

### ChatGPT-Enhanced ROC Analysis (CERA) web tool

The proposed CERA web tool, freely available at https://datascicence.shinyapps.io/ROCGPT/, is developed using the shiny app [27], pROC [8] R package and *cutpointr* function [28]. There are three versions of CERA tool. In version 1 and 2, it calculates optimized cutoff values for the *X* continuous variable (biomarker) and the *Y* binary outcome variable. In addition to providing the ROC curve plot and the distribution of the biomarker, it also shows statistical results such as mean, mode, median, and standard deviation of the biomarker. It also visualizes the relationship between the biomarker and the binary response variable with a boxplot class distribution graph. Finally, CERA can report this information in three parts with ChatGPT-supported interpretations: Introduction, Results and Conclusion sections with Data Quality Checking, ROC Plot and Perfromance Measures subsections in Results section. The reporting is connected and data is retrieved via the ChatGPT, developed by OpenAI [29]. This data is converted into a Rmarkdown HTML format that can be downloaded by the user. The only difference between version 1 and version 2 is whether the ChatGPT-generated report is automatically developed. In version 1, users cannot modify the prompts used in report generation, whereas in version 2, they have the capability to write their own prompts. Additionally, in version 3, the questions asked by users are processed by ChatGPT to generate outputs in code or text format, which, if necessary, can be converted into graphical representations. In CERA Version 3, unlike Versions 1 and 2, ChatGPT-interpreted reports are not generated; however, users can download the input prompts and the outputs (logs) generated by ChatGPT.

### Workflow steps for version 1

**Fig 4:**
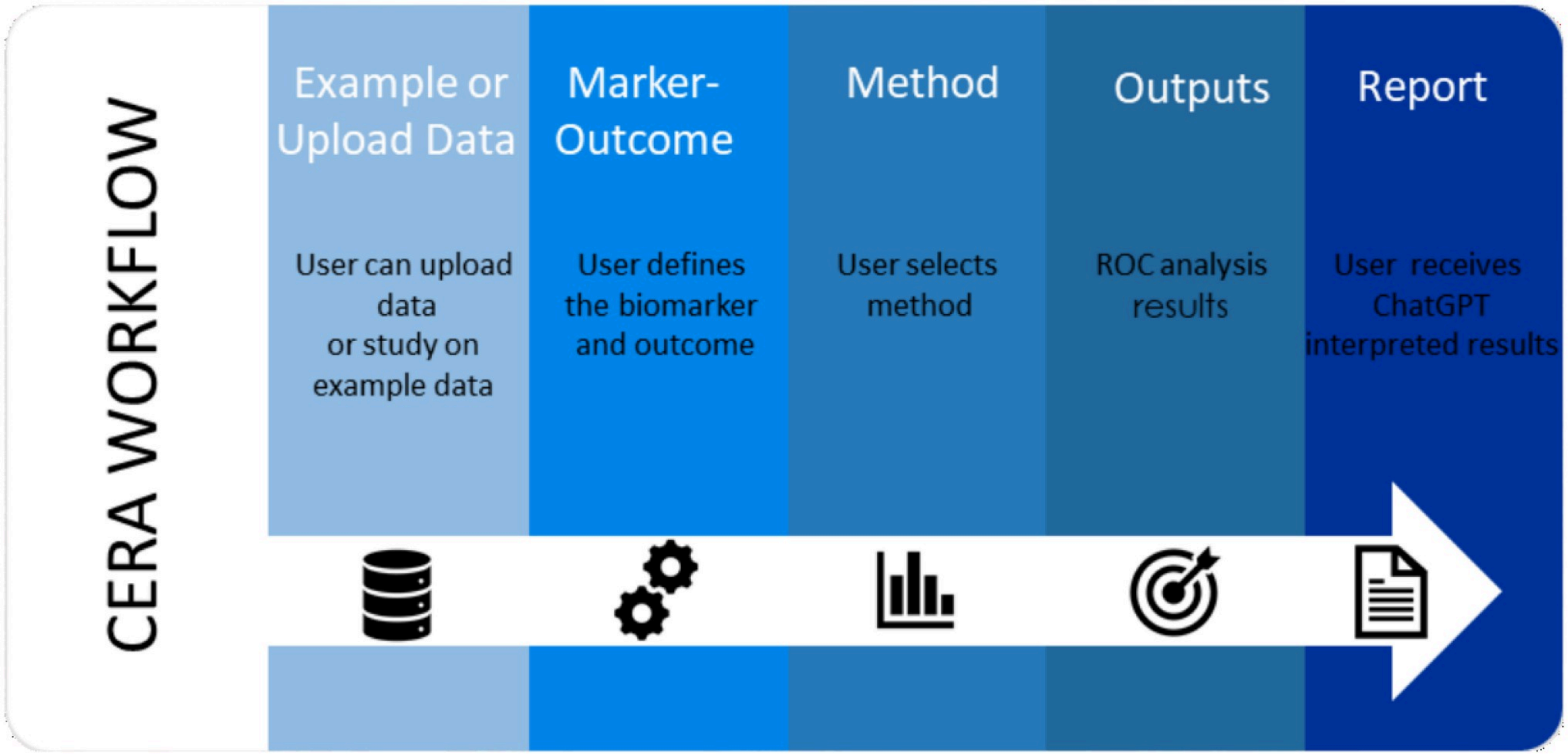
Workflow of the CERA web tool for ROC analysis for version 1. The CERA workflow consists of the following steps: Example or Upload Data: In this step, the user can upload their own data or example datasets provided by CERA tool to the system. Marker-Outcome: In this step the should select the biomarker and the outcome variable. Method: The user selects the method they want to apply in the study. Outputs: This is the step where ROC graph and performance characteristics are taken. Report: By clicking “Download Reprt”, the user can download the report prepared with ChatGPT under the sections of Intorduction, Results and Conclusion of the CERA outputs.

The workflow involved in data processing, as illustrated in Fig 4, is listed in the subsequent items:

1. **Example or Upload Data:** The user can upload data in a comma-separated format, with the header first row. Additionally, the user has the option to work with example datasets provided in the tool, including the Wisconsin breast cancer dataset [22], the PIMA dataset, the aSAH dataset, or synthetic non-alcoholic fatty liver disease (NAFLD) data.
2. **Marker-Outcome:** The user defines the biomarker and binary response from the uploaded data.
3. **Method:** The user selects the cutoff method from the Select Method part. The Select Method part has youden_topleft, cutoff, maximized and min_pvalue_approach.
  - youden_topleft: Users can select either the Youden Index (youden) method or the Closest Top-Left (closest.topleft) method under the “Select Index” conditional panel.
  - cutoff: Users can specify a specific cutoff value.
  - maximized: Users can select the minimum and maximum values for specificity and sensitivity under the “Select Constrain Metric” conditional panel. If users click on the ’Specificity’ option, then they need to specify the minimum specificity value under the ’Enter minimum constraint value’. The same process applies to sensitivity.
  - min_pvalue_approach: Users can select the minimum p-value approach.
4. **Outputs:** The CERA tool analyses the data, generates ROC curve plots and related results.
5. **Report:** There is a “Download Report” button to generate a ChatGPT generated report and receive the output in an HTML file.

The CERA tool performs data analysis and ROC curve analyses, with the main aim of selecting the optimal cutoff value. Understanding the nuances of selecting the appropriate cut-off value is essential for leveraging the full potential of diagnostic or predictive tests. Choosing the optimal cut-off value is a critical step when seeking to harness the full potential of a diagnostic or predictive test. It serves as the threshold where a test result transitions from one classification to another, for instance, from “negative” to “positive”. Its significance cannot be overstated, as the implications of such classifications can have profound impacts on subsequent medical or research decisions. Over the years, numerous criteria, primarily anchored in ROC analysis, have been put forward to determine the optimal cut-off value. The values on the ROC curve are the candidate cut-off value, and the most optimal one is determined by the tarde-off between Sensitivity and Specificity. Sensitivity represents the test’s ability to correctly identify true positives, while specificity represents its ability to correctly identify true negatives. In certain situations, Sensitivity takes precedence over Specificity [30], especially when dealing with highly contagious diseases or those with severe consequences. Conversely, there are scenarios where Specificity is favored over Sensitivity, particularly when follow-up diagnostic procedures are either hazardous or costly [31]. However, when neither Sensitivity nor Specificity is prioritized, an advisable strategy would be to optimize both metrics. Based on this information, it is imperative for the user to ascertain the most optimal cut-off value.

The cutoff methods used in the tool can be determined as follows.

- Youden’s Index: Youden’s index was first introduced by William John Youden [32] to optimize the cutoff value of the ROC curve. This optimization is done by maximizing the difference between sensitivity and (1-specificity), as represented by Youden’s statistics in the Eq 1.
  $$\begin{array}{c} J=\text{sensitivity}+\text{specificity}-1 \end{array}$$ (1)
  The range of Youden’s index is from -1 to 1. A value of 0 suggests that the performance of the classifier is equivalent to a random selection, whereas a value of 1 means perfect classification performance. As the value of *J* increases, the performance is deemed better, given that it implies a greater disparity between the true positive rate and the false positive rate. Youden’s index has a simple and intuitive interpretation: it corresponds to the point on the curve that is farthest from the “chance” line [33].
- Closest top-left: This method chooses the best cutoff threshold by identifying the value closest to the upper-left corner of the ROC curve as shown in Eq 2, to find the optimum cutoff in ROC curve analysis. This method calculates the optimal cutoff by the min value of the square distance between (0,1) and the value on the ROC curve, i.e.
  $$\begin{array}{c} d=min\{\sqrt{{\left(1-\text{sensitivity}\right)}^{2}+{\left(1-\text{specificity}\right)}^{2}}\}, \end{array}$$ (2)
  where *d* is calculated for each cutoff, and the cutoff that gives the minimum distances is selected as the optimal cutoff value. It is important to note that the top-left method, being a simple heuristic, may not consistently provide the optimal threshold for a given problem.
- Minimum p-value approach: Cutoff values are calculated by minimum p-value approach by creating a 2 × 2 table for each potential cutoff value and selecting the optimal cutoff, *c*, that maximizes the chi-square statistics or minimizes the *p* value. A disadvantage of the min-*p*-value approach is that it can result in a high number of Type I errors.
- Minimum sensitivity/specificity: The user can define minimum or maximum specificity or sensitivity values, and thus the defined value takes the minimum of that value. For example, if the user defines a value of 0.7 for specificity, the performance measure results show the value with a minimum specificity of 0.7. This method works on *cutpointr* function [28].
- Biomarker-oriented approaches: The tool automatically calculates the mean, median, and mode. These results can also be used as cutoff points in biomarker-oriented approaches.

In addition, the user can manually define the cutoff value and see the performance measures of it.

### Workflow steps for version 2

**Fig 5:**
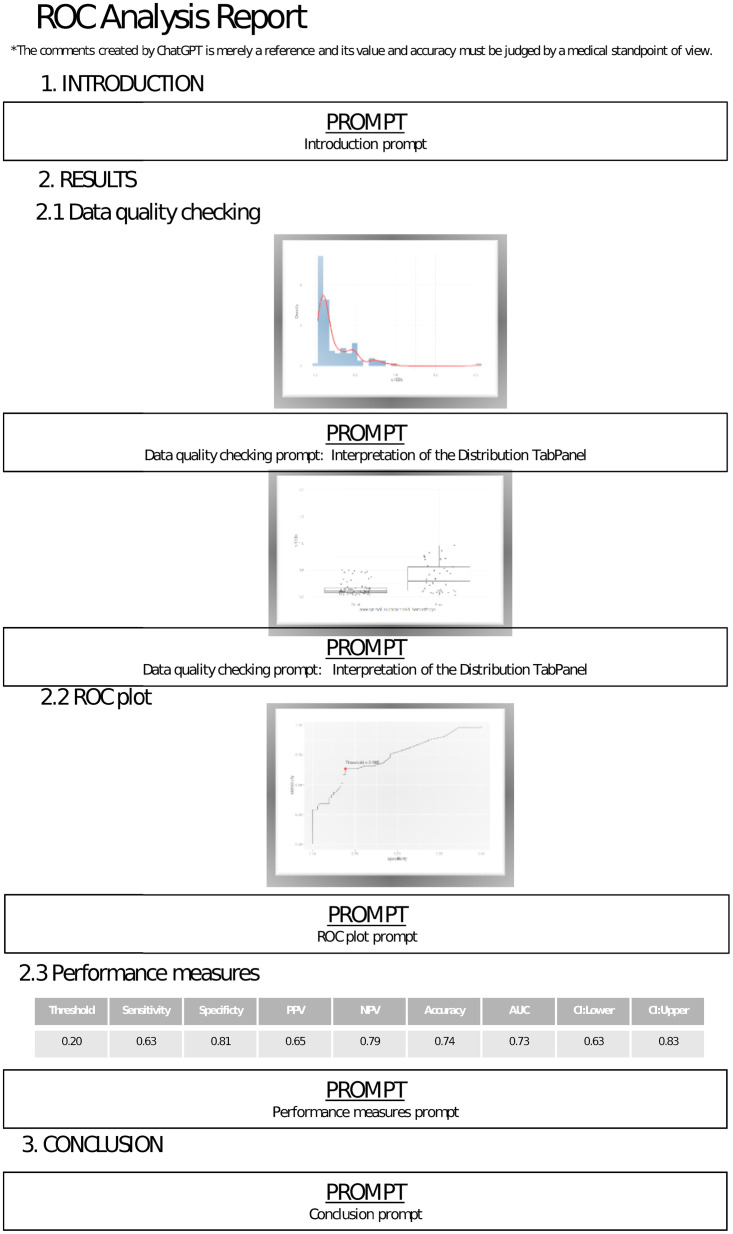
Prompt representation of fields for each section and subsection in the report generated by the CERA tool. **Introduction Prompt:** Users can write their own prompt based on their specific requirements. **Data Quality Checking Prompt—Interpretation of the Distribution TabPanel:** Users have the flexibility to write any prompt they desire to interpret the figures under the Distribution TabPanel. **Data Quality Checking Prompt—Interpretation of the Class Distributions TabPanel:** Users can create their own prompts to interpret the boxplot found under the ‘Class Distributions’ section. **ROC Plot Prompt:** Users are able to write their own prompt to interpret the ROC curve. **Performance Measures Prompt:** Users can compose their own prompt to interpret the performance measures displayed in the Table TabPanel. **Conclusion Prompt:** Users can write prompts related to the outcomes of their analysis.

In the default version, specifically Version 1, the only information that ChatGPT can receive pertains to the names of the variables in the uploaded datasets or in the example datasets. To address this limitation, we developed Version 2, allowing users to input additional information or craft their own prompts. In this enhanced version, users have the capability to formulate their own prompts for each section and subsection within the draft report depicted below the Fig 5.

Workflow of the CERA tool version 2 steps are represented below. In the following steps. Steps 1,2,3,4 and 6 are the same as in the workflow of version 1.

1. **Example or Upload Data:** The user can upload data in a comma-separated format, with the header first row. Additionally, the user has the option to work with example datasets provided in the tool, including the Wisconsin breast cancer dataset [22], the PIMA dataset, the aSAH dataset, or synthetic non-alcoholic fatty liver disease (NAFLD) data.
2. **Marker-Outcome:** The user defines the biomarker and binary response from the uploaded data.
3. **Method:** The user selects the cutoff method from the Select Method part. The Select Method part has youden_topleft, cutoff, maximized and min_pvalue_approach.
  - youden_topleft: Users can select either the Youden Index (youden) method or the Closest Top-Left (closest.topleft) method under the “Select Index” conditional panel.
  - cutoff: Users can specify a specific cutoff value.
  - maximized: Users can select the minimum and maximum values for specificity and sensitivity under the “Select Constrain Metric” conditional panel.
  - min_pvalue_approach: Users can select the minimum p-value approach.
4. **Outputs:** The CERA tool analyses the data, generates ROC curve plots and related results.
5. **Creating own prompts:** User can create their own prompts for each section and subsections in the report as seen in Fig 5.
  - **Introduction prompt:** Users can write their own prompts for the Introduction section. An example prompt could be: “Write an introduction paragraph of at least 2000 characters based on the information: We are conducting an ROC curve analysis for NAFLD disease. Our chosen biomarker is HSI, and the binary outcome under study is the presence or absence of NAFLD. We have obtained an AUC value of 0.86, and the optimal cutoff value has been identified as 45.03.”
  - **Data quality checking prompt (Interpretation of the Distribution TabPanel):** This prompt clarifies the distribution of the biomarker as showcased in the Distribution TabPanel. Therefore, users should craft their prompts based on the explanatory details of the biomarker. An example prompt can be: “We are conducting an ROC curve analysis on HSI in association with NAFLD. I will provide statistics such as the mean, standard deviation, and median for the HSI biomarker. Analyse the distribution of this variable. The mean stands at 43.12, the standard deviation at 12.32, with a range from a minimum value of 6.45 to a maximum of 82.96 and mode 448.31.”.
  - **Data quality checking prompt(Interpretation of the Class Distributions TabPanel):** In this prompt, users can describe the boxplot found under the ‘Class Distributions’ section of the tool. An example of such a prompt is: “We are studying the biomarker HSI in relation to a binary outcome variable. This binary outcome, represented by ‘NAFLD’, categorizes into two classes: NAFLD disease and non-NAFLD. For the predictor variable HSI related to NAFLD disease, the statistics are as follows: a mean value of 50.66, a minimum value of 21.54, and a maximum value of 82.78. In contrast, for the HSI values corresponding to non-NAFLD cases, the mean is 45.36, the minimum is 17.13, and the maximum is 72.1. Based on this information, interpret these two classes.”.
  - **ROC plot prompt:** In this part, users are encouraged to offer interpretations of the ROC plot. For instance, an example prompt could be: “We are undertaking an ROC curve analysis focusing on HSI in the context of NAFLD, achieving an AUC of 0.87. Provide your detailed interpretation of the ROC plot’s implications.”.
  - **Performance measures prompt:** In this section, users have the autonomy to interpret performance metrics by crafting their own prompts. An exemplar prompt might be phrased as: “Interpret the subsequent performance measures derived from the HSI in relation to NAFLD: threshold of 45.33, sensitivity at 0.73, specificity at 0.89, PPV at 0.87, NPV at 0.77, accuracy measuring 0.81, and AUC at 0.87.”.
  - **Conclusion prompt:** Users can craft personalized prompts tailored to their needs in this Conclusion section. Here’s an example: “In this investigation, a Receiver Operating Characteristic (ROC) curve analysis was conducted to ascertain the optimal cut-off points for the HSI biomarker in the context of NAFLD. Our chief aim was to pinpoint the most suitable cut-off values for the early detection and diagnostic processes of NAFLD. By utilizing these specific values, medical professionals can achieve a more accurate assessment of risks associated with HSI in their patients, ultimately culminating in enhanced patient outcomes and precise therapeutic strategies. Notably, the study reveals an AUC of 0.87 and establishes a threshold of 45.32 for the HSI biomarker. Based on this data, please formulate a comprehensive conclusion paragraph spanning at least 1000 characters.”.
6. **Report:** There is a “Download Report” button to generate a ChatGPT generated report and receive the output in an HTML file.

### Workflow steps for version 3

**Fig 6:**
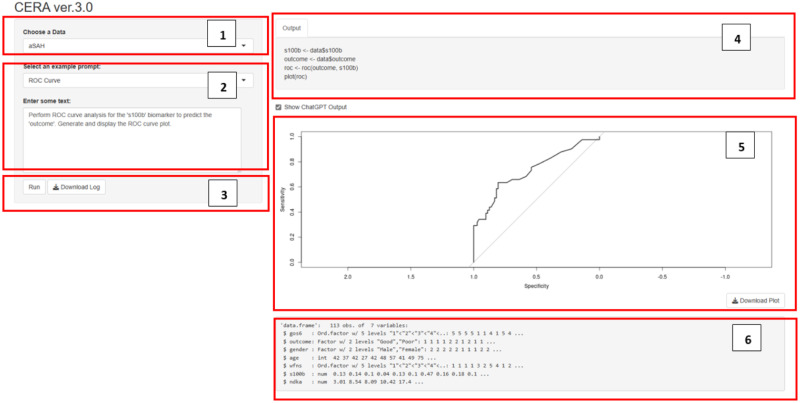
CERA ver.3.0 interface showcasing a workflow for ROC curve analysis, with Example Datasets and Data Upload part (1), User Input option (2), Run and Download Logs button (3), Code Output (4), Graphical Representation of results (5) and Data Structure (6).

We have released the version 3 which is the final version of the CERA tool. This version generates code based on the user prompts and plot the generated code. User not only can get a code or plot this code but also can receive text outputs. The user interface of the CERA version 3 can be represented in Fig 6.

The workflow of the tool represented in Fig 6 is as follows:

1. Example Datasets and Data Upload: Users can either upload their own data or use the example datasets provided by various R libraries. In addition to the datasets, aSAH and PIMA, which were used in version 1 and 2, the following datasets are included:
  - cancer dataset: Derived from the survival package [34], it captures data from one of the initial successful trials of adjuvant chemotherapy for colon cancer with 228 observations and 10 variables.
  - alzheimer dataset: Obtained from the modeldata library [35], Craig-Schapiro et al. [36] chronicle a study involving 333 patients. This included individuals with mild cognitive impairment as well as healthy subjects. The aim was to differentiate subjects in early stages of impairment from those who are cognitively healthy.
  - actg315raw dataset: Sourced from the ushr library [37], this dataset includes data from the ACTG315 clinical trial of HIV-infected adults undergoing ART. Details of 46 individuals with 361 observations are documented, detailing HIV viral load measurements observed on specified days for up to 28 weeks post-treatment initiation.
  - heart dataset: From the kmed library [38], this mixed-variable dataset comprises 14 variables relating to the diagnosis of heart disease in 297 patients.
  - tumor dataset: Extracted from the dobson library [39], it presents tumor responses of total 16 male and female patients undergoing treatment for small-cell lung cancer.
  - ILPD dataset: From the ExNRuleEnsemble library [40], the dataset includes 416 liver patient records and 167 non-liver patient records, collected from the northeast of Andhra Pradesh, India. Patients are categorized into two groups based on their liver condition.
2. User Input: Users ask questions. Additionally, we provide example prompts for each dataset. For example, for the aSAH dataset, we suggest: “Perform ROC curve analysis for the ‘s100b’ biomarker to predict the ‘outcome‘. Generate and display the ROC curve plot.” Based on these prompts, the tool will generate the corresponding code or text.
3. Run and Download Logs: Users can run the analysis by clicking on the Run button. Users can also download the log file. The log file includes the asked questions and generated codes/text by ChatGPT.
4. Code Output: This section displays the code or text produced by ChatGPT. If users wish to view this output, they can select the “Show ChatGPT Output” radiobutton.
5. Graphical Representation: The tool visualizes the data based on the generated code.
6. Data Structure: Users can view the structure of the data.

### External validation of proposed method on Regensburg Pediatric Appendicitis (RPA) data

**Fig 7:**
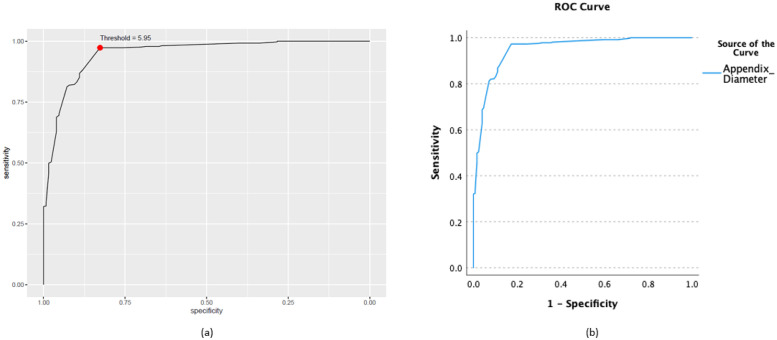
Comparison of ROC curves using the CERA tool and SPSS for the Regensburg Pediatric Appendicitis (RPA) dataset, focusing on the diameter of appendix in relation to diagnosis (Appendicitis vs. no appendicitis). The ROC curve plots are derived from the CERA tool (a) and SPSS (b) applied to the RPA dataset.

In our study, we validated the CERA tool using the publicly available Regensburg Pediatric Appendicitis (RPA) dataset [41], which was acquired from pediatric patients with abdominal pain at Children’s Hospital St. Hedwig in Regensburg, Germany, including diverse ultrasound images and comprehensive clinical data. After excluding missing values and retaining only the records labeled as ‘appendicitis’ (371 patients) and ‘no appendicitis’ (127 patients), we obtained a total of 498 observations. For this purpose, we conducted a ROC analysis on the RPA dataset, focusing on the diameter of appendix in relation to diagnosis (appendicitis vs. no appendicitis). We used three different statistical analysis platforms for this analysis: SPSS, R (employing the easyROC package [12]), and the CERA tool. We then closely compared the outcomes of these analyses, as presented in Table 2. It was observed that the results obtained from the CERA tool were in perfect agreement with those from R and SPSS. This included identical values for key statistical metrics: the AUC at 0.95, Youden’s Index at 5.95 (mm), a specificity of 0.83, and a sensitivity of 0.97. Daldal and Dagmura [42] showed that the diameter of the appendix is a significant factor in diagnosing acute appendicitis. According to their study, an appendix diameter greater than 6 mm increases the likelihood of appendicitis. Our result, at 5.95 mm, closely approaches this threshold. Furthermore, we also conducted a visual analysis by plotting the ROC curve using both SPSS and the CERA tool. These comparative visual results are illustrated in Fig 7. This comprehensive analysis demonstrates the reliability and accuracy of the CERA tool in analyzing RPA data.

**Table 2:** Comparative analysis of ROC curve results using SPSS, R (easyROC), and the CERA tool for the Regensburg Pediatric Appendicitis (RPA) dataset: Focusing on the diameter of appendix in relation to diagnosis (Appendicitis vs. no appendicitis).

Tool	AUC	Threshold (Youden’s Index)	Specificity	Sensitivity
CERA	0.95	5.95	0.83	0.97
SPSS	0.95	5.95	0.83	0.97
R (easyROC)	0.95	5.95	0.83	0.97

## Results and discussion

**Fig 8:**
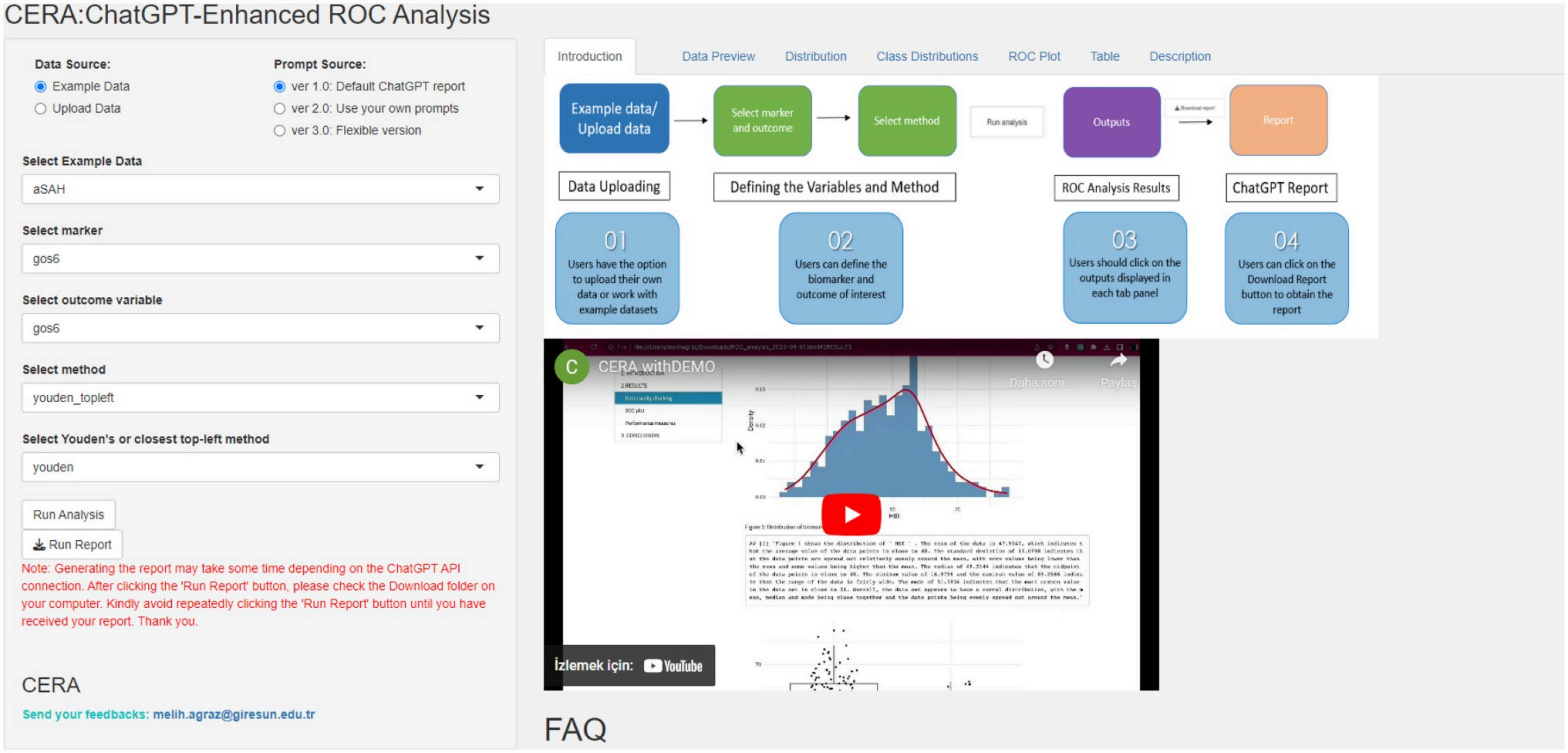
Uploading data and using example datasets in the CERA tool. The figure shows the process of uploading data and using example datasets within the CERA tool. The CERA tool has four example datasets: three publicly available (Wisconsin breast cancer, PIMA, aSAH) and one simulated dataset (NAFLD) derived from real data. Users have the option to select one of these example datasets or upload their own data. For uploaded data, it should be in comma-separated format with the first row serving as the header containing variable names.

In order to demonstrate the effectiveness of our CERA tool, we use four example distinct datasets: Wisconsin breast cancer dataset [22], ii) PIMA dataset, iii) aSAH dataset and synthetic iv) simulated NAFLD dataset. The user can use the provided example datasets or have the option to upload their own data. It is important to note that user-uploaded data must contain a header row with variable names. This data can be uploaded by selecting the Upload Data radio button as seen in Fig 8.

After the data is uploaded, the user can run the analysis by clicking on “Run analysis” button. The CERA tool then automatically analyses the data, presenting the results in six different Tab panels in version 1 and 2. These panels are:

- Data Preview: Shows the first and the last 4 observations of the dataset, number of observations, number of labels in the output and percentage of missing data.
- Distribution: This panel shows the distribution of the biomarker, along with statistical analysis of the biomarker such as mean, median, and standard deviation.
- Class Distribution: This panel provides a boxplot of the labels of biomarkers.
- ROC Plot: This panel brings the ROC curve of the analysis.
- Table: This panel provides performance measures of the output such as cutoff value, sensitivity, specificity, positive predictive value (PPV), negative predictive value (NPV), accuracy, AUC, lower bound of the AUC confidence interval, and upper bound of the AUC confidence interval.
- Description: This panel offers brief information about the method used.

**Fig 9:**
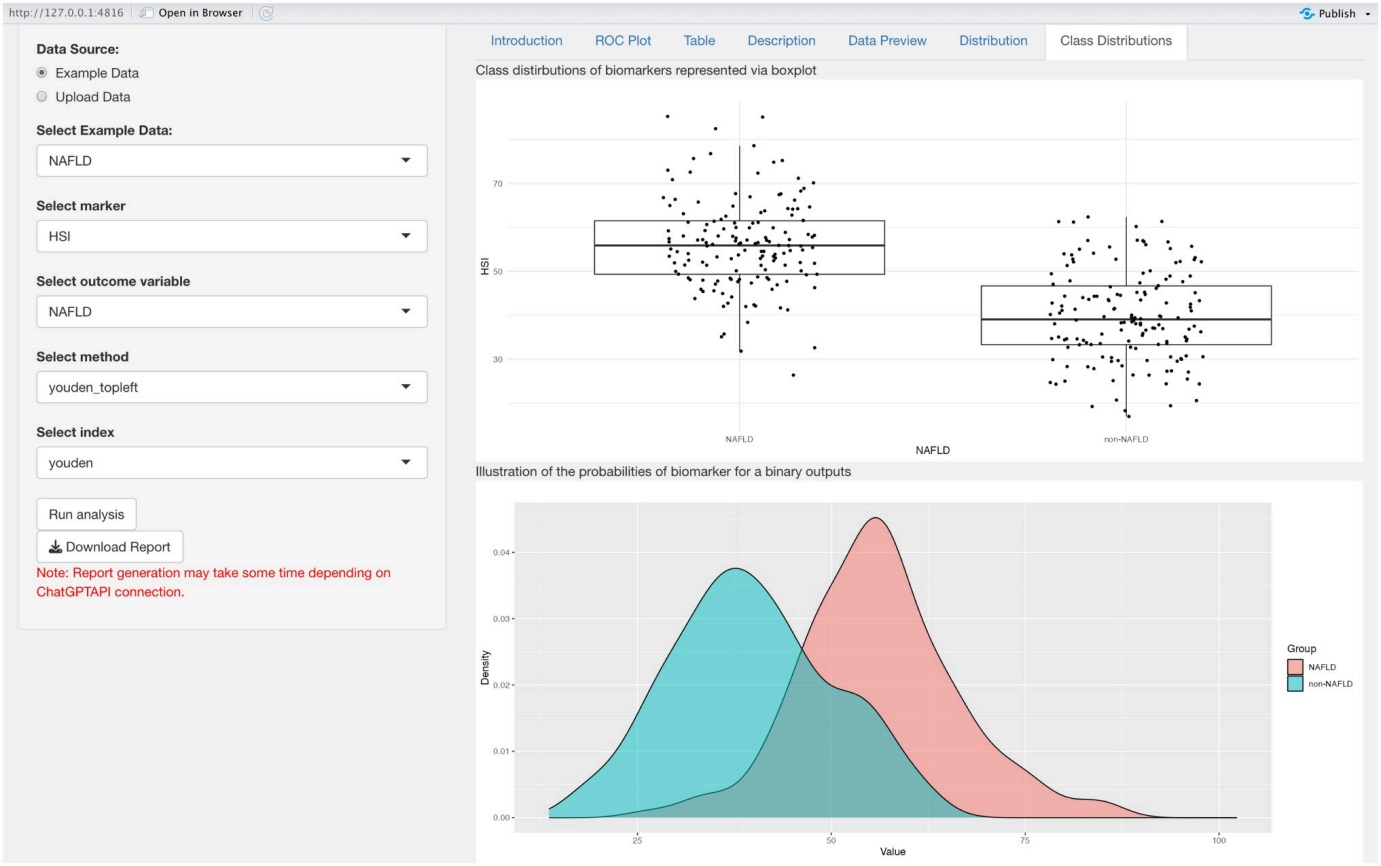
Class distributions of biomarker. The figure illustrates the class distributions of biomarkers using boxplots. In the top figure, the boxplot displays the class distributions of biomarkers. In the example of NAFLD, the mean value is around 50, while the non-NAFLD value is around 40. This indicates a noticeable difference between the NAFLD and non-NAFLD classes. In the bottom figure, users can obtain probability distributions of the biomarker for a binary outcome. In the example, it is observed that the NAFLD and non-NAFLD classes exhibit some degree of overlap.

**Fig 10:**
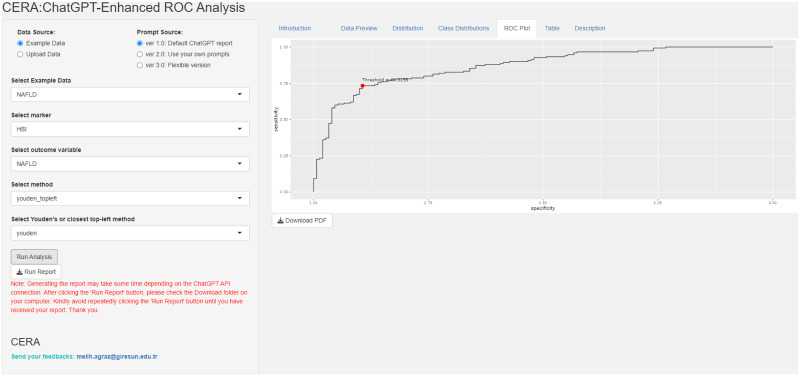
ROC plot output of the CERA tool. ROC Plot generated by the CERA tool. This graph illustrates the relationship between specificity and sensitivity, offering a visual representation of a classification model’s effectiveness via the ROC curve. In this depicted instance, the curve suggests an AUC value greater than 0.5. For precise AUC values, users are encouraged to consult the Tables panel within the CERA tool. The optimal cutoff value is also highlighted.

The “**Data Preview**” tab panel lists the first and last four rows of the dataset. In addition, users can access the number of observations, number of labels in the output, and missing data percentages under the following titles “Number of Observations”, “Number of Labels”, “Percentage of Missing Data” (%), respectively. The “**Distribution**” panel is dedicated to visualizing the distribution of a selected biomarker. It features two primary components: a histogram and a density plot. Additionally, the panel provides essential statistical insights about the biomarker: its mean value, the standard deviation, and the median. The “**Class Distribution**”, as depicted in Fig 9, tab panel focuses on visualizing how the selected biomarker is distributed across different predefined classes or groups. The graphical representation employs box plots, a type of chart that provides a summary of a dataset’s distribution, including its central value, variability, and potential outliers. The Y-axis represents the biomarker’s values, while the X-axis categorizes data into various classes. Each box plot illustrates the central tendency, spread, and potential outliers of the biomarker values within each class. This visualization aids in comparing the distribution of the biomarker across the different classes and identifying any discernible patterns or trends. In Fig 9, the upper panel titled ‘Class distributions of biomarkers represented via boxplot’ shows the biomarker distribution for two different groups: NAFLD and non-NAFLD. The boxplots reveal that the NAFLD group has a higher median value and a wider interquartile range, indicating greater variability within this group. Additionally, there are more outliers above the upper whisker, suggesting that higher biomarker values are more prevalent in the NAFLD group compared to the non-NAFLD group. The lower panel, labeled ‘Illustration of the probabilities of biomarker for a binary outputs’, presents density plots for both groups. These plots provide a visual comparison of the biomarker’s distribution in a continuous format. The overlapping area indicates the range of values where both conditions exhibit similar biomarker levels. The “**ROC Plot**” tab panel, as seen in Fig 10, provides a graphical representation of the ROC curve. By analyzing the curve, users can evaluate the discriminatory power of a diagnostic test or marker. Additionally, the curve highlight an optimal threshold point specified by users before the analysis that balances sensitivity and specificity, aiding in decision-making regarding the utility of the test or marker in question. The ROC plot in the Fig 10 is a visual tool used for evaluating the diagnostic accuracy of a test for NAFLD, where ‘HSI’ stands for Hepatic Steatosis Index, a biomarker for the NAFLD. The curve represents the trade-off between sensitivity and specificity at various threshold settings. The marked red point on the curve suggests the optimal cutoff determined by Youden’s index, providing the best balance between sensitivity and specificity for the test. Table 3 provides the performance measures as depicted in the “**Table**” panel of the CERA tool. As represented in the example results, the tool calculates a threshold of 0.20 and an AUC of 0.73 (with confidence intervals ranging from confidence interval lower boundary (CI:Lower B.) 0.63 to confidence interval upper boundary (CI:Upper B.) 0.83). Additionally, it computes sensitivity, specificity, positive predictive value (PPV), negative predictive value (NPV), and accuracy. The “**Description**” panel provides explanations for the selected methods. These definitions are static and are not generated by ChatGPT in each iteration.

**Table 3:** Performance measures of ROC curve analysis. The table represents the performance measures of ROC curve analysis, as shown in the Table Tab panel of the CERA tool. The results indicate the following metrics: threshold: 0.20, Sensitivity: 0.63, Specificity: 0.81, Positive Predictive Value (PPV): 0.65, Negative Predictive Value (NPV): 0.79, Accuracy: 0.74, Area Under the Curve (AUC): 0.73, Confidence Interval (CI) of Lower Boundary (LB)-Upper Boundary (UB): 0.63-0.83. These performance measures provide valuable insights into the accuracy and effectiveness of the ROC curve analysis performed by the CERA tool.

Threshold	Sensitivity	Specificity	PPV	NPV	Accuracy	AUC	CI: LB-UB	
0.20	0.63	0.81	0.65	0.79	0.74	0.73	0.63	0.83

**Fig 11:**
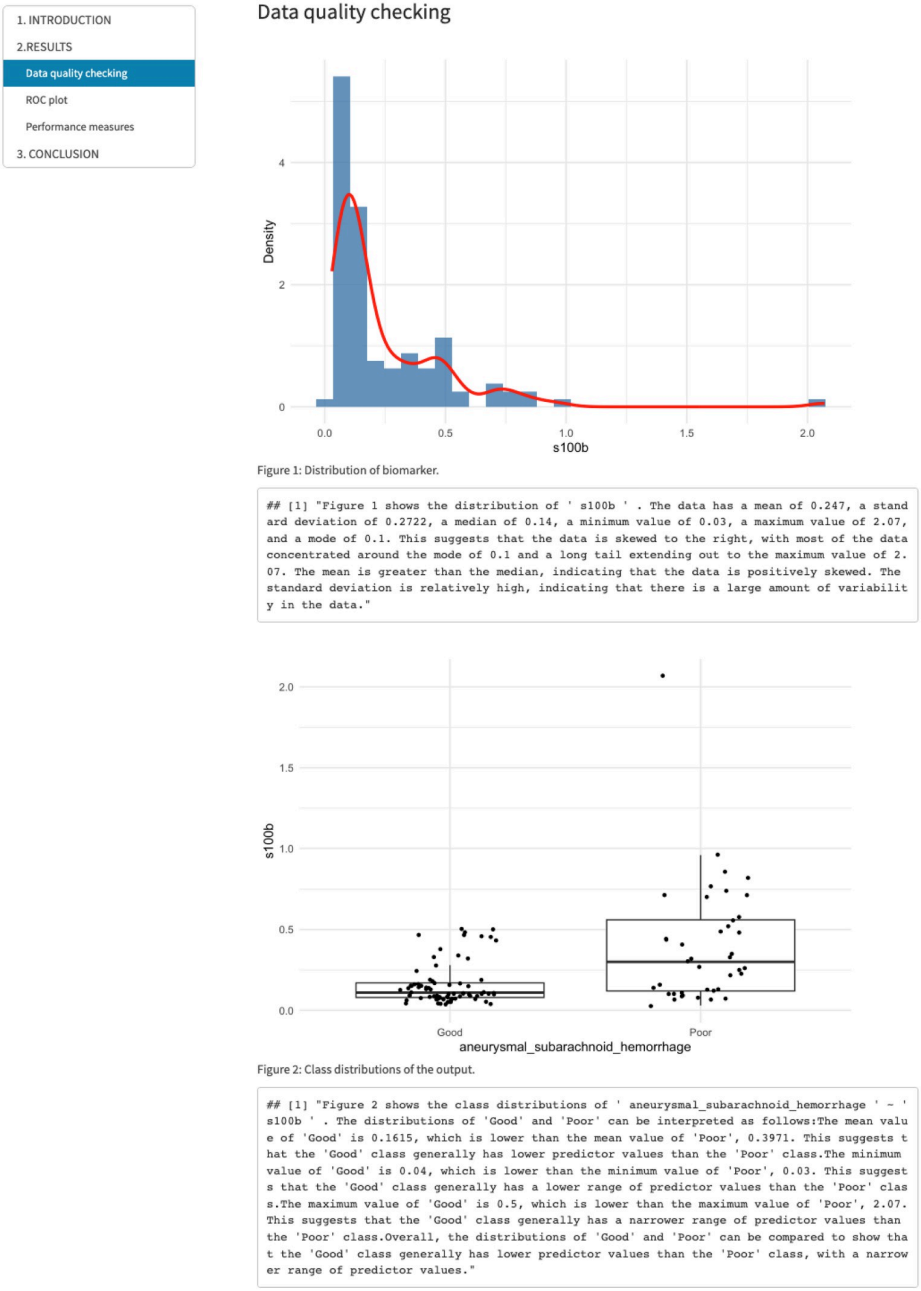
Report generated by CERA tool via ChatGPT in version 1 and 2—Data quality checking subsection of RESULTS section. The figure displays a subsection titled “Data Quality Checking” within the RESULTS section of the report generated by the CERA tool using ChatGPT. This subsection includes figures from the Class Distribution panel, accompanied by interpreted results from ChatGPT. In the top figure, the distribution of the biomarker is illustrated, with a red distribution curve overlaid to provide additional information. This visualization aims to understand the distribution pattern of biomarkers. The bottom figure presents the class distributions of the output variable in the form of a boxplot that updates as ChatGPT interprets the results. According to this figure, aneurysmal subarachnoid hemorrhage data with binary class (Poor/Good) is visualized. The average value of s100b in the Poor-grade aneurysmal subarachnoid hemorrhage class is higher than the mean value of s100b in the Good-graded aneurysmal subarachnoid hemorrhage class.

After generating results using the CERA tool in version 1 and 2, users may require a comprehensive interpretation and report. To facilitate this, we have included a “Download Report” button that produces a report enriched by ChatGPT, as shown in Fig 11. The generation of the report could take up to 3 minutes, depending on the ChatGPT API connection; when it is ready, the user can find it in their Download folder. We are working on optimizing this process to make it more efficient and faster. This report is divided into three sections: Introduction, Results, and Conclusion, each of which is prepared via the ChatGPT API connection [29].

- In the **Introduction** section, it is strongly advised that users utilize appropriate and descriptive column names for the biomarker and outcome variables instead of abbreviations in version 1. This is crucial because the column names are used as input by ChatGPT to generate interpretations. By using clear and informative column names, it enhances the accuracy and understanding of the generated interpretations. However, if the dataset does not have clear variable names, or if the user wants to add extra information into the prompts, they can prefer version 2.
- The **Results** section contains three subsections: Data Quality Checking, ROC Plot, and Performance Measures. The Data Quality Checking subsection includes the distribution plot generated in the “Distribution” tab panel, which is interpreted based on the statistics of the biomarker. Additionally, the “Class Distribution” tab panel plot and its interpretation are included. In the ROC Plot subsection, users can find the ROC curve generated in the “ROC Plot” tab panel along with its interpretation. The Performance Measures subsection features the results from the “Table” tab panel and their interpretations.
- Finally, the **Conclusion** section concisely explains the outputs and summarizes the results.

**Fig 12:**
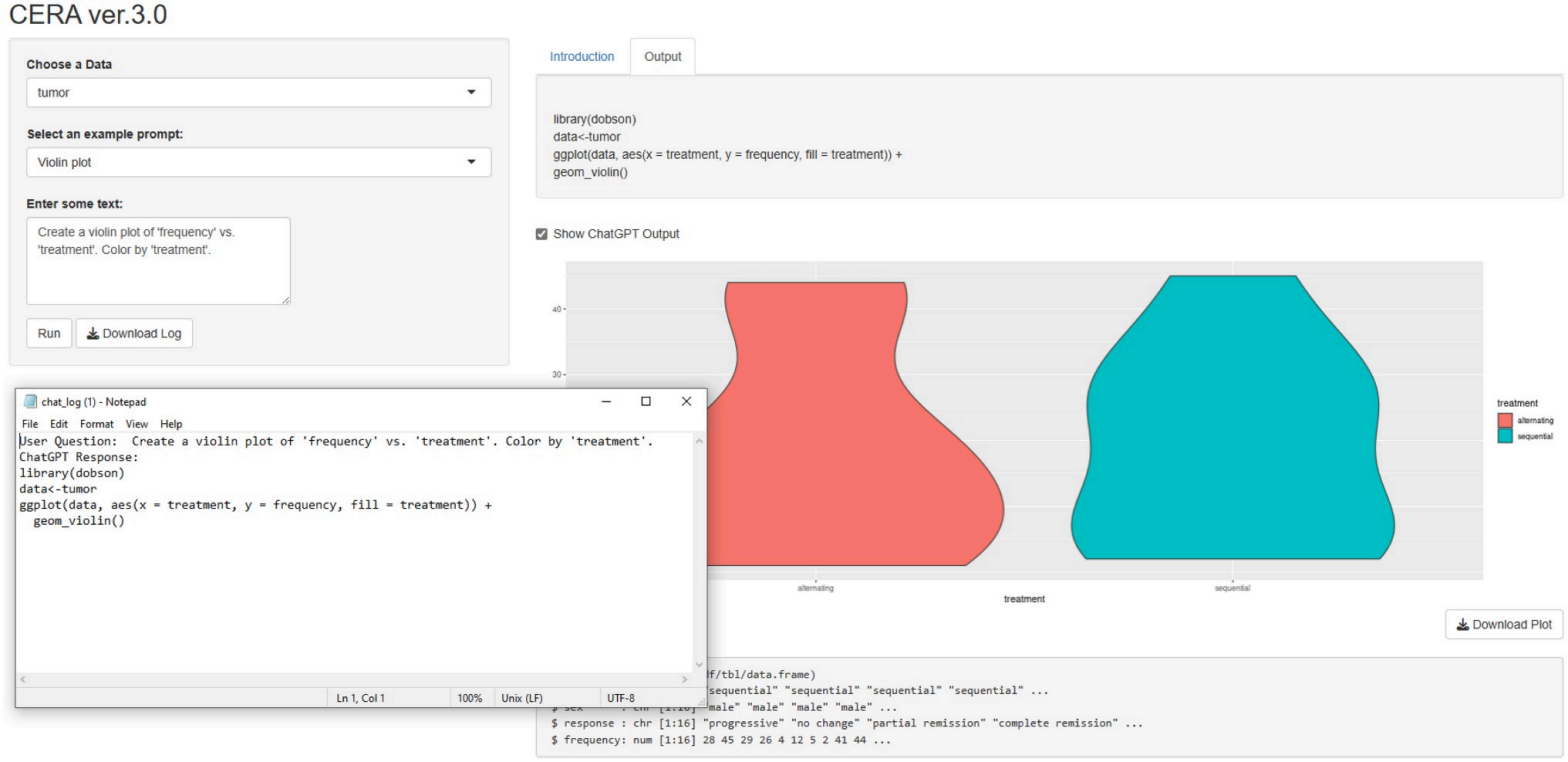
The CERA version 3.0 interface. The tool allows users to input specific plot requests. In this example, a violin plot visualizing ‘frequency’ against ‘treatment’ types–‘alternating’ and ‘sequential’–is generated. Users can also download logs of their inputs along with the ChatGPT-generated outputs.

Since versions 1 and 2 were primarily useful for data analysis and ROC curve analysis, we aimed to make the CERA tool more flexible. Consequently, we developed version 3, as illustrated in Fig 12. As previously explained, CERA version 3 takes user questions and converts them into code/text outputs, and generates graphs if required. Additionally, the violin plot in the Fig 12 shows the distribution of a frequency variable for two different treatments, ‘alternating’ and ‘sequential’, within a dataset related to tumor treatment. Each ‘violin’ represents one treatment group, with the thickness of the plot indicating the density of data points at different frequency levels. The wider sections of the violin show where data is more concentrated, signifying a higher occurrence of those frequency values. From the plot, it can be inferred that the ‘sequential’ treatment group has a more concentrated range of frequency values, as indicated by the pronounced peak, while the ‘alternating’ treatment group displays a broader spread of frequency values, suggesting a more varied response to treatment.

## Discussion

ROC curve analysis is an important tool in determining the performance of biomarkers in diagnostic tests. In this study, we introduce a web-based shiny tool called CERA (ChatGPT-Enhanced ROC Analysis), designed to facilitate ROC curve analysis available at https://datascicence.shinyapps.io/ROCGPT/. This tool allows users to easily perform ROC analysis, as well as interprets and reports findings to the user via ChatGPT in the Introduction, Results and Conclusion sections in version 1 and 2. In the CERA tool in version 1 and 2, users have the option to upload their own data or use one of the provided datasets. After the data is uploaded, users define the biomarker and can choose from various optimization methods. The tool generates ROC curve graph and optimized cutoff value, as well as additional performance metrics. It also provides various statistical results regarding the biomarker. In the final step, users can generate an interpreted report with ChatGPT. In addition, we developed CERA version 3, which allows users greater flexibility in data analysis compared to versions 1 and 2.

ROC curve analysis can be performed using various tools such as SPSS, Minitab or Stata or programming languages such as R and Python. However, in this work, we introduce an open source, freely accessible web tool developed with shiny app. This tool has a user-friendly interface and provides many statistics necessary for data analysis, including ROC analysis. The feature that distinguishes this tool from others is that it can interpret outputs via ChatGPT and can generate detailed reports that users can use in their work in version 1 and 2.

It is important to note that the CERA tool does not store user data in any database and we cannot guarantee data security. The tool only works through the hosting platform powered by shiny.io. In addition, since ChatGPT may provide inaccurate information, we cannot guarantee that the interpretations in the report are correct. In future work, we plan to expand the capabilities of CERA to support multiple biomarkers. We also aim to include additional data preprocessing steps such as missing data imputation or outlier detection. We believe these improvements will enable this tool to serve as a more effective platform for data science purposes.
